# Simulation of Winter Wheat (*Triticum aestivum* L.) Response to Saline Irrigation Using AquaCrop in the Tadla Plain, Morocco: Implications for Irrigation Management

**DOI:** 10.3390/plants15121899

**Published:** 2026-06-18

**Authors:** Khadija Manhou, Rachid Moussadek, Abdelmjid Zouahri, Zoubida Belmahi, Majda Oueld Lhaj, Hatim Sanad, Hasna Yachou, Driss Hmouni, Houria Dakak

**Affiliations:** 1 Laboratory of Natural Resources and Sustainable Development, Department of Biology, Faculty of Sciences, Ibn Tofail University, Kenitra 14000, Morocco; 2 Research Unit on Environment and Conservation of Natural Resources, Regional Center of Rabat, National Institute of Agricultural Research, AV. Ennasr, Rabat 10101, Morocco; 3 International Center for Agricultural Research in the Dry Areas (ICARDA), Rabat 10100, Morocco; 4 Research Unit on Plant Breeding, Conservation and Valorisation of Phytogenetic Resources, Regional Center of Agricultural Research Rabat, National Institute of Agricultural Research (INRA), Rabat 10000, Morocco; 5 Laboratory of Process Engineering and Environment, Faculty of Science and Technology Mohammedia, University Hassan II of Casablanca, Mohammedia 28806, Morocco

**Keywords:** AquaCrop, saline irrigation, winter wheat, biomass, grain yield, soil salinity

## Abstract

Saline irrigation is increasingly practiced in semi-arid regions to cope with freshwater scarcity; however, it strongly affects crop growth, water use, and soil salinity. This study aims to calibrate and validate the AquaCrop model to simulate key growth parameters of winter wheat (cv. Achtar) under saline irrigation conditions in the Tadla Plain, Morocco, focusing on canopy cover (CC), actual evapotranspiration (ETa), soil water content (SWC), biomass (B), and grain yield (GY). The model was first calibrated using observed data from the 2023 growing season and subsequently validated using data from the 2022 growing season. Overall, AquaCrop effectively reproduced crop growth during both calibration and validation phases. During calibration, canopy cover was accurately simulated, with average RMSE values below 1%, while biomass and grain yield were also well reproduced, with low RMSE values (0.25 t ha^−1^ for B and 0.10 t ha^−1^ for GY), confirming the robustness of the calibrated parameters. The model also performed well in simulating ETa and SWC, capturing the seasonal dynamics of crop water use and soil moisture. During validation, ETa was satisfactorily reproduced, with an RMSE of approximately 0.80 mm day^−1^, while SWC showed good agreement with observations, with NRMSE values ranging from 7.9 to 10.5%. Grain yield and biomass were reliably predicted, with NRMSE values below 4%. These results demonstrate that AquaCrop is a reliable tool for simulating winter wheat under saline irrigation and for assessing crop response under salt-affected conditions, providing an integrated evaluation of crop performance, water use, and soil salinity dynamics to support improved irrigation management and water-use efficiency under semi-arid conditions.

## 1. Introduction

In arid and semi-arid regions, agriculture faces increasing challenges due to limited freshwater availability and progressive soil salinization. These constraints are further intensified by high evaporation rates, prolonged droughts, and the preferential allocation of high-quality water resources to urban and industrial sectors. Soil salinization, driven by both natural processes and anthropogenic activities such as inefficient irrigation, poor drainage, and excessive fertilizer use, negatively affects soil structure, fertility, plant development, and overall crop productivity [1,2]. Global population growth, projected to reach 9.7 billion by 2050, further intensifies the challenge of ensuring sufficient, safe, and nutritious food for all [3]. Currently, agriculture accounts for approximately 70–75% of global freshwater withdrawals, placing significant pressure on water resources [4]. However, the expansion of water use in agriculture is expected to be limited to around 10%, emphasizing the urgent need for efficient water management strategies directly applicable at farm level. In this context, the use of alternative water sources, such as saline groundwater, drainage water, or treated wastewater, represents a potential solution to sustain agricultural production. Nevertheless, these practices introduce additional challenges, including salt accumulation, increased soil sodicity, and degradation of soil physical properties. These effects can reduce root development, limit water infiltration, and compromise long-term soil fertility [5,6,7]. Therefore, developing site-specific irrigation strategies that maintain crop productivity while minimizing soil degradation is essential. Climatic variability, characterized by irregular rainfall patterns, high evapotranspiration, and the increasing frequency of extreme weather events, further exacerbates water scarcity and soil salinization, posing serious threats to crop productivity and food security.

Winter wheat (*Triticum aestivum* L.) is a staple crop of global importance, providing more than 20% of the calories consumed worldwide and representing a key economic resource, particularly in Mediterranean and semi-arid regions [8,9]. Wheat grains are widely used in the production of bread, pastries, and other food products of high nutritional and commercial value. However, wheat is moderately sensitive to salinity, especially during critical growth stages such as germination, tillering, stem elongation, and grain filling, where stress can significantly reduce yield and quality [10]. Saline or inadequate irrigation can negatively affect yield components, including spike density, grain filling, and thousand-grain weight, as well as grain quality traits such as protein content and gluten strength [11,12]. These impacts can be mitigated through optimized irrigation management that considers both water quantity and quality. Moreover, additional environmental stresses such as drought and high temperatures further influence crop growth, water productivity, and yield stability [13,14,15]. In arid and semi-arid environments, where rainfall satisfies only a limited portion of crop water requirements, irrigation becomes essential to achieve optimal yields [16,17]. However, intensive use of groundwater has caused declining water tables, threatening the sustainability of production. Therefore, strategic use of marginal, saline, or brackish water becomes a practical solution to sustain wheat productivity while conserving high-quality freshwater, highlighting the vital importance of water availability and quality for winter wheat cultivation in water-limited areas [18].

To optimize winter wheat production under limited water availability and salinity stress, field experiments remain essential. They enable the evaluation of different management strategies and provide a better understanding of the interactions between soil, plants, and the atmosphere, as well as their effects on growth, yield, and stress tolerance [19,20,21]. Winter wheat, being moderately salt-tolerant, has a soil salinity threshold of approximately 6 dS m^−1^ and an irrigation water salinity threshold of around 4 dS m^−1^, beyond which yield can be significantly reduced. This moderate tolerance necessitates the implementation of management practices that specifically aim to limit salt accumulation in the root zone in order to sustain productivity [22,23,24,25]. Deficit irrigation using slightly saline water, carefully adjusted to the crop’s critical water requirements, has been shown to be more effective than excessive irrigation, which can reduce water use efficiency and cause nutrient losses [26,27,28]. Rainfall, which influences both salt leaching and crop performance, should also be considered when planning irrigation [29].

Among the tools available for simulating crop growth and water use, the FAO-developed AquaCrop model is particularly notable for its capacity to predict winter wheat yield, biomass, canopy cover, and water productivity under both water-limited and saline irrigation conditions [30]. Crop growth simulations depend on complex interactions among climate, soil characteristics, plant traits, and management practices, and many models require extensive input data that are often unavailable, limiting their practical applicability. AquaCrop is especially valuable because it integrates soil water and solute transport processes with historical climate data, allowing the development of practical irrigation strategies under variable rainfall and salinity conditions [31,32]. By employing a water-driven approach combined with an integrated solute transport routine, AquaCrop allows for the assessment of soil salinity dynamics and the effects of salt stress on plant development using a relatively small set of intuitive parameters. Compared with more complex models such as SWAP, HYDRUS, or SALTMED, it offers a balanced combination of simplicity, accuracy, and robustness, facilitating model calibration, validation, and the design of site-specific irrigation strategies that are directly applicable under real farming conditions [33,34,35,36].

AquaCrop has been widely applied across various crops and environmental settings, demonstrating its robustness in simulating crop growth, yield, and water productivity under water-limited and saline conditions [37,38]. Previous studies have provided valuable insights into model performance, irrigation management, and crop response to salinity across different agro-ecological contexts [39,40,41]. These contributions have significantly improved the understanding and application of AquaCrop for optimizing agricultural water use. However, while these studies clearly demonstrate the capabilities of the model, fewer studies have explored a comprehensive evaluation of crop growth dynamics and soil–water processes under real field conditions based on multi-variable datasets. In particular, the combined use of field-based calibration, independent validation without parameter adjustment, and multi-year simulations under contrasting climatic conditions remains relatively limited. Further integration of these approaches would enhance the applicability of AquaCrop under diverse and variable farming conditions. In this context, the present study provides a comprehensive assessment of AquaCrop performance under combined salinity and semi-arid conditions. The model is calibrated using detailed field measurements, including canopy cover, soil water content, evapotranspiration, biomass, and grain yield, and validated using an independent dataset without parameter adjustment. In addition, the integration of field observations with multi-year simulations enables a robust evaluation of model performance under variable environmental conditions and supports the development of practical irrigation strategies aimed at improving water-use efficiency while minimizing soil salinity risks.

Building upon this approach, the study integrates field-based calibration and validation with multi-year AquaCrop simulations to improve saline irrigation management under water-limited and salt-affected conditions. Particular emphasis is placed on ensuring the practical applicability of the modeling approach by incorporating realistic irrigation practices and salinity levels representative of field conditions. This framework enables a more accurate assessment of crop response under combined water and salinity stresses. Therefore, the specific objectives of this study were to: (i) calibrate the AquaCrop model under controlled saline irrigation conditions using field data covering a wide and realistic gradient of irrigation water salinity levels; (ii) validate the model using field observations to assess its predictive performance under varying salinity and water stress conditions; (iii) analyze the coupled interactions between crop growth, water use, and soil salinity dynamics by simultaneously evaluating plant and soil responses; (iv) apply the validated model to perform multi-year simulations under contrasting climatic conditions in order to assess model robustness under variable environmental conditions; and (v) identify salinity tolerance thresholds of the studied cultivar and develop practical, field-applicable irrigation strategies aimed at improving water-use efficiency while minimizing soil salinity risks.

## 2. Materials and Methods

### 2.1. Field Site Description and Experimental Design

**Figure 1:**
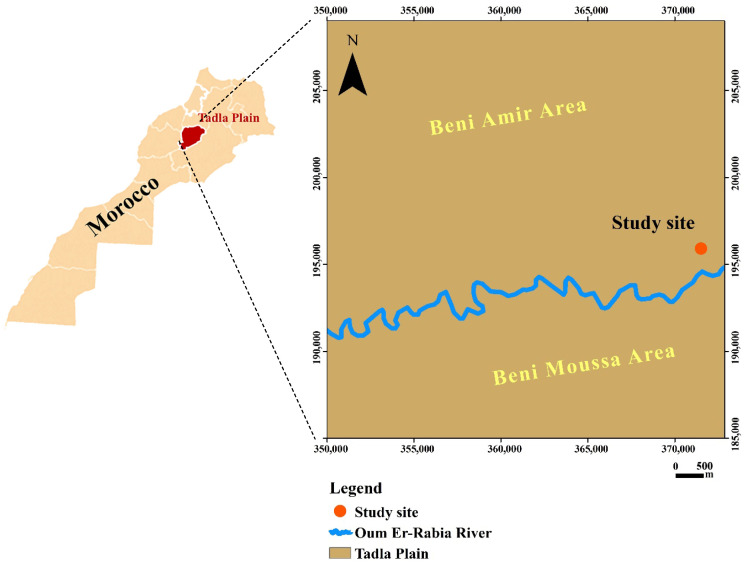
Location of the study area in the Tadla plain, central Morocco.

The Tadla Plain is a major WNW–ESE–oriented synclinal depression located in central Morocco, covering nearly 3600 km^2^ between 32°28′49″–32°31′10″ N and 6°42′21″–6°16′03″ W (Figure 1) [42,43]. The basin is bordered to the north by the gently rising Phosphate Plateau and to the south by the Jurassic formations of the High Atlas Mountains. Toward the east, the plain narrows along the Oum Er-Rbia River as it approaches the rugged Zaian uplands, while its western extent merges gradually with the Bahira region; the lower reach of the El Abid River is commonly considered the hydrogeological boundary. It stretches approximately 125 km in length and up to 50 km in width, with elevations ranging from 350 to 500 m. The lowest point (315 m) is located at the Sidi Driss hydrological station along the Oum Er-Rbia [44].

The Beni Amir irrigated perimeter has undergone substantial agricultural intensification since 1954, supported by fertile soils and reliable surface-water resources, making it one of the most productive sectors of the Tadla irrigation scheme [45]. The region experiences a semi-arid Mediterranean climate with marked seasonal contrasts. Rainfall is concentrated in winter and early spring, while a prolonged dry period typically spans from late May to mid-autumn. Long-term climatic observations indicate an average annual precipitation of about 393 mm [46], with March and April registering the highest monthly totals. Summers are extremely hot, with peak temperatures approaching 38 °C, whereas winter nighttime temperatures may fall close to freezing. Atmospheric evaporative demand remains high throughout most of the year, reaching a maximum in July and August and declining substantially during winter. Reference evapotranspiration exceeds 1000 mm per year, reflecting the strong climatic water deficit characteristic of the area. The field experiment was conducted on an experimental area of 33 m × 35 m, arranged according to a randomized complete block design (RCBD) with four replicates to account for spatial variability within the field. The soil of the experimental site was preliminarily surveyed to ensure relative homogeneity in texture and topography prior to plot establishment. Five irrigation water salinity treatments were evaluated: I0 (1.5 dS m^−1^), I2 (3 dS m^−1^), I3 (5 dS m^−1^), I4 (7 dS m^−1^), and I5 (9 dS m^−1^). These treatments were randomly assigned within each block to avoid positional bias and ensure unbiased comparisons among salinity levels. Each experimental plot measured 5 m in length, and plots were separated by 3 m-wide buffer alleys to minimize lateral movement of salts and water between adjacent treatments and to facilitate field operations. Buffer zones were carefully maintained throughout the experiment to prevent cross-contamination among salinity treatments. Winter wheat was sown uniformly across all plots using identical row spacing and seeding density to ensure consistent crop establishment and growth conditions. All agronomic practices, including fertilization, weed control, and pest management, were applied uniformly across treatments following local agricultural practices, so that irrigation water salinity remained the only experimental factor [47,48].

### 2.2. Data Collection

#### 2.2.1. Climatic Data

Meteorological measurements, including maximum and minimum air temperatures (Tmax and Tmin), mean temperature, wind speed (measured at 2 m height), relative humidity, solar radiation, and precipitation, were obtained from the CRAT meteorological station located in the Beni Amir irrigated area, approximately 9 km east of Fquih Ben Salah within the Tadla irrigated perimeter, Morocco, at 32°28′08″ N, 06°41′24″ W and 434 m asl, and operated by the Regional Office for Agricultural Development of Tadla (ORMVAT). These data were used to calculate reference evapotranspiration (ET_0_) using the FAO Penman–Monteith equation [49].

**Figure 2:**
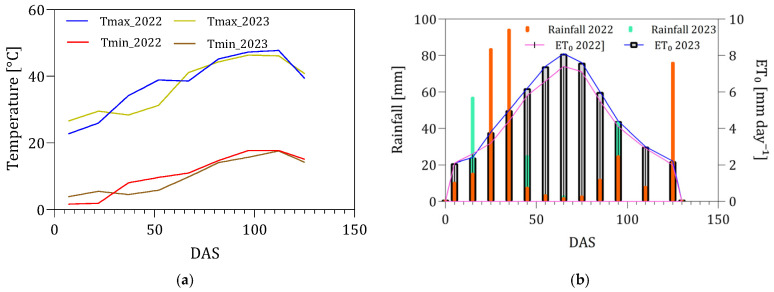
Seasonal variations in (**a**) daily maximum and minimum air temperatures (Tmax and Tmin) and (**b**) reference evapotranspiration (ET_0_) and monthly precipitation during the 2022 and 2023 growing seasons.

Figure 2 presents the time series of ET_0_, air temperature, and rainfall for the calibration (2023) and validation (2022) seasons. Daily ET_0_ values ranged from approximately 2.1 to 8.1 mm day^−1^ during the calibration season and from 2.0 to 7.4 mm day^−1^ during the validation season, indicating a higher atmospheric evaporative demand in 2023. Thermal conditions differed between the two seasons. The calibration season recorded maximum air temperatures up to 47.8 °C and minimum temperatures between 3.9 and 17.6 °C, whereas the validation season exhibited slightly lower maximum temperatures (up to 46.3 °C) and minimum temperatures ranging from 1.6 to 17.7 °C. These differences suggest greater thermal stress during the calibration season, particularly during the mid- to late-growth stages, which may affect crop development and evapotranspiration dynamics.

Rainfall was low and irregular in both seasons but differed in distribution. The 2023 season received less than 200 mm with sparse events, whereas 2022 recorded higher rainfall exceeding 200 mm, with more frequent early-season precipitation, likely improving soil moisture availability and crop water uptake. In addition, long-term climatic data used for scenario analysis were obtained from the NASA POWER database [50], which provides open-access satellite-derived climatic data widely used in scientific research.

#### 2.2.2. Soil Data

Soil data used in the AquaCrop model were obtained from laboratory analyses of soil samples collected from the experimental field located in the Tadla irrigated perimeter prior to sowing. Sampling was carried out to characterize the initial soil conditions of the study site. A total of 12 soil samples were collected randomly across the field at three depths (0–30, 30–60, and 60–90 cm) and homogenized to form composite samples for each layer. The soil profile used in AquaCrop was parameterized based on these samples. For each layer, particle-size distribution, pH, electrical conductivity (ECe), organic matter content, calcium carbonate, mineral nitrogen (NH_4_^+^ and NO_3_^−^), cation exchange capacity (CEC), bulk density, and hydraulic properties were determined.

Soil samples were air-dried at room temperature to reach a stable moisture state, gently disaggregated, and sieved to <2 mm. A subsample of this fraction was further reduced to <0.2 mm for chemical analyses. Particle-size distribution was determined using the sedimentation method after chemical dispersion, allowing the quantification of sand, silt, and clay fractions [51]. Soil pH was measured potentiometrically using a glass-electrode pH meter (Mettler Toledo, Columbus, OH, USA) [52], while soil electrical conductivity was determined on the saturated paste extract using a calibrated conductivity meter (Orion model 162, Thermo Scientific, Waltham, MA, USA) [53]. Organic matter content was quantified following the Walkley–Black wet oxidation method [54], total nitrogen was determined using the Kjeldahl procedure [55], and CEC was measured by ammonium acetate extraction (1 N NH_4_OAc) [56]. These measured soil properties were subsequently used to derive the hydraulic parameters required for model simulations, including saturated water content (θsat), field capacity (FC), permanent wilting point (PWP), and saturated hydraulic conductivity (Ksat), ensuring consistency between soil texture, bulk density, and water retention characteristics within the modeling framework.

In addition to the initial characterization, soil samples were collected at four key phenological stages of winter wheat (tillering, stem elongation, anthesis, and physiological maturity) to monitor temporal changes in soil moisture and salinity. Soil water content (SWC, %) was monitored using the gravimetric method. The volumetric soil water content (θ, m^3^ m^−3^) was calculated by multiplying the gravimetric water content by the soil bulk density.

The soil parameters presented in Table 1, derived from the initial soil characterization, were used as input data for the AquaCrop model during calibration and simulation.

#### 2.2.3. Crop Management

Winter wheat (cv. Achtar) was sown using a mechanical seed drill at a density of 300 seeds m^−2^ to ensure uniform crop establishment across all experimental plots. The experiment was conducted using experimental plots arranged in a randomized complete block design (RCBD) with four replications, allowing controlled application of irrigation and salinity treatments. Immediately after sowing, all plots were well irrigated to promote seed emergence and early seedling growth. The study was conducted over two growing seasons, with 2023 used for calibration and 2022 for validation. The cultivar was selected for its adaptability to irrigated systems, moderate tillering capacity, and semi-early to semi-late maturity cycle, making it suitable for the agroclimatic conditions of the study area (Table 2). Fertilization was applied uniformly across all treatments to avoid nutrient-induced variability, with a basal application of 200 kg ha^−1^ of triple superphosphate and 100 kg ha^−1^ of potassium sulfate at sowing, and a total nitrogen input of 150 kg ha^−1^ split into three applications (60, 30, and 60 kg N ha^−1^ at sowing, tillering, and stem elongation, respectively). Crop protection practices were uniformly applied across seasons. The crop reached physiological maturity and was harvested in mid- to late June in both 2022 and 2023. All agronomic practices were kept consistent between seasons to ensure that differences in crop performance were primarily attributable to irrigation water salinity (Table 3).

#### 2.2.4. Irrigation Management

Irrigation was applied using a gravity-fed system supplied by a 3000 L tank to ensure uniform water distribution across all plots. Each 15 m^2^ plot received 1500 L per irrigation event, corresponding to an irrigation depth of 100 mm (1000 m^3^ ha^−1^). Irrigation volume and application method were kept identical across treatments so that irrigation-water salinity remained the only experimental factor. Freshwater used for irrigation was groundwater abstracted from the Béni Amir irrigated perimeter and served as the control treatment (1.5 dS m^−1^). Five irrigation-water salinity levels (1.5, 3, 5, 7, and 9 dS m^−1^) were selected to reflect the spatial and temporal variability of irrigation water salinity in the Béni Amir area, where electrical conductivity ranges from 1.83 to 9 dS m^−1^, with the 2–4 dS m^−1^ class being the most prevalent. This range therefore encompasses both commonly used irrigation waters and higher salinity conditions that may occur locally due to groundwater abstraction, water mixing, and water scarcity periods. Saline irrigation waters were prepared by adding controlled amounts of sodium chloride (NaCl) to the same groundwater source until the target electrical conductivity levels (3, 5, 7, and 9 dS m^−1^) were reached, ensuring consistent and reproducible salinity conditions across irrigation events. Electrical conductivity was measured prior to each irrigation event using a calibrated conductivity meter to maintain consistency among treatments.

Irrigation during the 2022 and 2023 growing seasons was applied on four fixed dates (5 December, 28 January, 3 March, and 16 April), corresponding to key phenological stages of winter wheat and aligned with regional agronomic practices. Using the same irrigation dates for all treatments ensured equal water supply and allowed the effect of salinity to be clearly assessed. The chemical characteristics of the irrigation water used in each treatment are summarized in Table 4 and were used as input data in the AquaCrop model to simulate osmotic stress and soil salinity dynamics [58].

### 2.3. Description of the AquaCrop Model

The AquaCrop model, developed by the FAO [37,38,59], is a water-driven crop model used to simulate crop growth, biomass production, and yield under different environmental and management conditions. In this study, simulations were performed using AquaCrop version 7.1, released in August 2023. The model is based on the relationship between crop transpiration and biomass accumulation through a normalized water productivity parameter (WP), while grain yield is estimated using the harvest index (HI). Crop development is described using canopy cover (CC), which evolves from emergence to a maximum value and declines during senescence. The canopy cover curve, defined by the initial canopy cover (CC_0_), canopy growth coefficient (CGC), maximum canopy cover (CCx), and canopy decline coefficient (CDC), is used to partition reference evapotranspiration into soil evaporation and crop transpiration.

To calculate transpiration, the model employs the equation:

(1) $$\mathrm{Tr}\;=\;K_{S\;}\times \;(K_{\mathrm{cTr},x\;\;}\times \;{CC}^{*})\;\times \;{\mathrm{ET}}_{0\;}$$

where Tr represents crop transpiration, K_cTr,x_ denotes the maximum crop transpiration coefficient, CC* signifies the canopy cover (%), Ks denotes the stress coefficient, and ET_0_ represents reference evapotranspiration.

The final above-ground dry biomass is estimated using the following equation:

(2) $$B\;=\;{WP}^{*}\times \;\sum T_{r}$$

where B is the final above-ground dry biomass (t ha^−1^), WP* is the normalized water productivity (g m^−2^), and ∑T_r_ is the cumulative actual crop transpiration over the growing season (mm).

AquaCrop calculates dry grain yield using the following equation:

(3) GY = B × HI

where B represents the final above-ground dry biomass (t ha^−1^) and HI denotes the harvest index.

**Figure 3:**
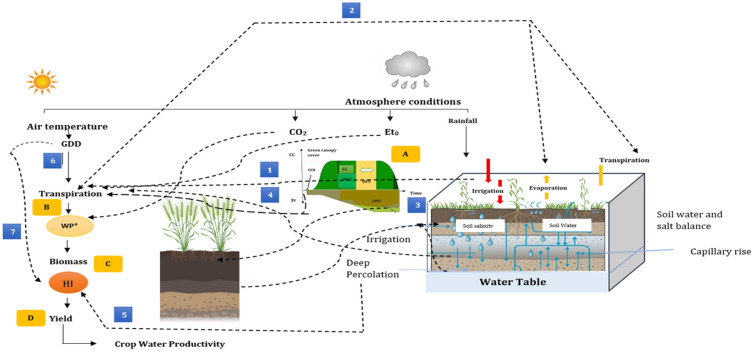
Calculation scheme of the AquaCrop model, showing the four sequential steps (A–D) and the associated processes (dotted arrows) affected by water stress (1–5) and temperature stress (6–7). CC is the green canopy cover; Zr is the rooting depth; CGC is the canopy growth coefficient; CDC is the canopy decline coefficient; GDD is the growing degree days; ET_0_ is the reference evapotranspiration; WP is the normalized biomass water productivity; and HI is the harvest index. Water stress: (1) slows canopy expansion, (2) accelerates canopy senescence, (3) decreases root deepening (only under severe stress), (4) reduces stomatal conductance and transpiration, and (5) affects the harvest index. Cold temperature stress (6) reduces crop transpiration, while extreme temperature stress (7) inhibits pollination and reduces HI [30].

Figure 3 illustrates the overall functioning of the AquaCrop model, showing the interactions between canopy cover, transpiration, biomass, and the harvest index, as well as the dynamics of soil water and salinity. Dashed arrows indicate processes influenced by water and heat stress, water availability, and atmospheric CO_2_ concentration.

### 2.4. Model Calibration and Validation

Calibration and validation are essential steps in model evaluation, as they reduce uncertainties and ensure that the model adequately represents the behavior of the system [60]. Model calibration involves identifying a set of parameters that best describe the system by comparing simulated outputs with observed data. Subsequently, model validation assesses the predictive capability of the model by evaluating its performance against independent observations [61]. In this study, the AquaCrop model was calibrated to simulate the growth, biomass production, and grain yield of winter wheat under the specific agro-climatic and irrigation conditions of the study area. At the initial stage of model parameterization, conservative parameters were retained at their default AquaCrop values, as they represent general crop characteristics and are not site-specific. Phenological development was defined based on field observations and expressed in growing degree days (GDD), which provide a robust representation of crop development under variable temperature conditions. In accordance with AquaCrop settings for wheat, a base temperature of 0 °C and an upper temperature of 26 °C were used. The timing of emergence, maximum canopy development, flowering, and the onset of senescence were derived directly from field observations, while the time to maturity was expressed in GDD and derived from the observed crop duration (Table 5).

The calibration procedure initially focused on canopy development, as canopy cover (CC) directly controls transpiration and biomass accumulation. Parameters governing canopy growth, including the initial canopy cover (CC_0_), maximum canopy cover (CCx), canopy growth coefficient (CGC), and canopy decline coefficient (CDC), were adjusted to ensure good agreement between simulated and observed canopy cover dynamics. Subsequently, crop parameters influencing transpiration and biomass production were calibrated. The maximum crop transpiration coefficient (K_cTr,x_), normalized crop water productivity (WP*), and reference harvest index (HI_0_) were fine-tuned using an iterative trial-and-error approach to minimize discrepancies between simulated and observed biomass (B) and grain yield (GY), as recommended in AquaCrop applications [37]. The maximum effective rooting depth (Zr) was also calibrated to improve the simulation of soil water content (SWC) and actual evapotranspiration (ETa).

Soil water balance parameters, including curve number (CN) and readily evaporable water (REW), were maintained at their default values, as they are considered conservative parameters. Similarly, soil water depletion thresholds (P_exp_, P_sto_, and P_sen_) and salinity response parameters were not modified. In contrast, salinity stress thresholds were adjusted to 5 and 18 dS m^−1^ to reflect local soil and irrigation conditions. Initial soil water content (SWC) was determined from field measurements prior to sowing and used as an input for model simulations, while temporal SWC measurements were used for calibration and validation.

Model calibration was performed by comparing simulated and observed values of canopy cover (CC), soil water content (SWC), actual evapotranspiration (ETa), total dry biomass (B), and grain yield (GY), until satisfactory agreement was achieved. For the validation phase, the calibrated parameter set obtained from the 2023 growing season was applied without further adjustment to the 2022 dataset. The selection of the calibration (2023) and validation (2022) seasons was based on data completeness and independence rather than chronological order. The 2023 dataset provided the most comprehensive measurements for robust calibration, while the 2022 dataset was used as an independent dataset to evaluate model performance under contrasting climatic conditions. As AquaCrop is a process-based model, its performance is not strictly dependent on the chronological order of the datasets. Model performance was evaluated by comparing simulated and observed CC, SWC, ETa, B, and GY, thereby assessing the robustness and predictive capacity of the AquaCrop model under the studied conditions.

### 2.5. Model Evaluation

**Figure 4:**
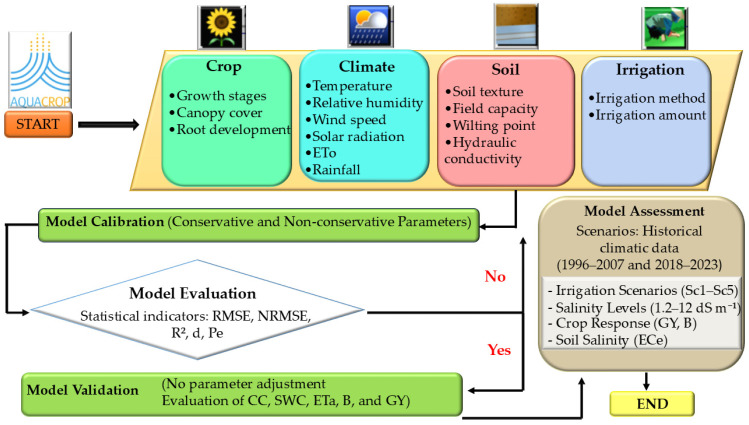
Flowchart of the AquaCrop model for simulating winter wheat.

This study employed several statistical metrics to evaluate the performance of the AquaCrop model, including percent error (Pe, Equation (4)), root mean square error (RMSE, Equation (5)), normalized root mean square error (NRMSE, Equation (6)), the coefficient of determination (R^2^, Equation (7)) [62], and the index of agreement (d, Equation (8)). These indicators were used to assess the agreement between observed (Oi) and simulated (Pi) values of canopy cover (CC), soil water content (SWC), actual evapotranspiration (ETa), grain yield (GY) and biomass (B).

(4) $$\mathrm{Pe}\;=\;\left(\frac{\mathrm{Pi}-\mathrm{Oi}}{\mathrm{Oi}}\right)\;\times \;100$$

(5) $$\mathrm{RMSE}\;=\;\sqrt{\frac{1\;}{n}}\sum_{i=1}^{n}\;{\left(\mathrm{Oi}-\mathrm{Pi}\right)}^{2}$$

(6) $$\mathrm{NRMSE}\;=\;\frac{\mathrm{RMSE}\;}{Ō}\;\times \;100$$

(7) $$R^{2}={\left[\frac{\sum_{i=1}^{n}\left(\mathrm{Oi}\;-\;O^{-})(\mathrm{Pi}\;-\;P^{-}\right)}{\sqrt{\sum_{i=1}^{n}(\mathrm{Oi}\;-\;Ō{)}^{2}}\;\times \;\sqrt{\sum_{i=1}^{n}(\mathrm{Pi}\;-\;P^{-}{)}^{2}}}\right]}^{2}$$

(8) $$d=1-\frac{\sum_{i=1}^{n}(\mathrm{Pi}\;-\;\mathrm{Oi}{)}^{2}\;}{\sum_{i=1}^{n}(\mathrm{Pi}\;-\;\mathrm{Oi})\;+|\mathrm{Oi}\;-P^{-}\;|\;{)}^{2}}$$

where Pi and Oi denote the simulated and observed values, respectively; P^−^ and O^−^ represent their corresponding means; and n is the total number of observations. These statistical indicators were used to quantify how well the simulated outputs matched the measured data during the calibration process. The evaluation metrics consisted of the coefficient of determination (R^2^), which measures the quality of fit between observed and simulated datasets; the root mean squared error (RMSE), which expresses the average magnitude of the deviation between simulated and observed values; and the normalized root mean squared error (NRMSE, %), calculated as the ratio of RMSE to the mean of the observed dataset. In addition, the index of agreement (d) was used to assess the overall degree of agreement between simulated and observed values, with values approaching 1 indicating better model performance. Additionally, the relative error (Pe) was employed to identify whether the model systematically underestimates or overestimates the observations. According to the NRMSE classification, model performance is considered excellent (<10%), good (10–20%), fair (20–30%), or poor (>30%) [63]. More detailed descriptions of these statistical metrics are provided in [64] and [65]. Figure 4 illustrates the schematic representation of the AquaCrop model input structure and workflow.

### 2.6. Description of Irrigation Salinity Management Scenarios

After model calibration and validation, AquaCrop was used to analyze winter wheat performance and soil salinity dynamics under a range of irrigation and salinity conditions combined with contrasting climatic periods (1996–2007 and 2018–2023). To account for inter-annual rainfall variability, each simulation year was classified as dry (P < 200 mm), normal (200 ≤ P ≤ 260 mm), or wet (P > 260 mm), based on long-term rainfall variability in the study area. Irrigation scheduling was based on regional agronomic recommendations and consistent with the management applied during the 2023 field experiment (calibration dataset), ensuring coherence between observed and simulated conditions. Five irrigation scenarios (Sc1–Sc5) were defined to represent increasing irrigation intensity across wheat phenological stages, with each irrigation event supplying 100 mm of water. Specifically, Sc1 corresponds to a highly deficit irrigation strategy with a single irrigation applied at the jointing stage, while Sc5 represents full irrigation with five events applied before winter, at jointing, booting, flowering, and grain filling stages. Intermediate scenarios (Sc2–Sc4) represent progressively increasing irrigation inputs. For each irrigation scenario, seven irrigation-water salinity levels were considered, defined as I0 (1.5 dS m^−1^), I1 (3 dS m^−1^), I2 (5 dS m^−1^), I3 (7 dS m^−1^), I4 (9 dS m^−1^), I5 (10 dS m^−1^), and I6 (12 dS m^−1^). These levels reflect the range of water quality sources commonly used in the Tadla region, including canal water, groundwater, and mixed or drainage water. This scenario-based approach was designed to systematically explore a wide range of crop responses under combined water and salinity stresses. By varying irrigation intensity and salinity levels across different climatic conditions, the simulations enabled the identification of trade-offs between crop productivity (grain yield and biomass) and soil salinity (ECe). Model outputs, including simulated grain yield (GY), biomass (B), and soil electrical conductivity (ECe), were analyzed to assess the combined and interactive effects of irrigation management, salinity, and climate variability.

This approach, applied after successful model validation using independent data from the 2022 growing season, allowed a comprehensive evaluation of the response of the Achtar wheat cultivar to realistic irrigation practices under semi-arid conditions.

## 3. Results

### 3.1. Calibration of AquaCrop Model

#### 3.1.1. Simulation of Canopy Cover

**Figure 5:**
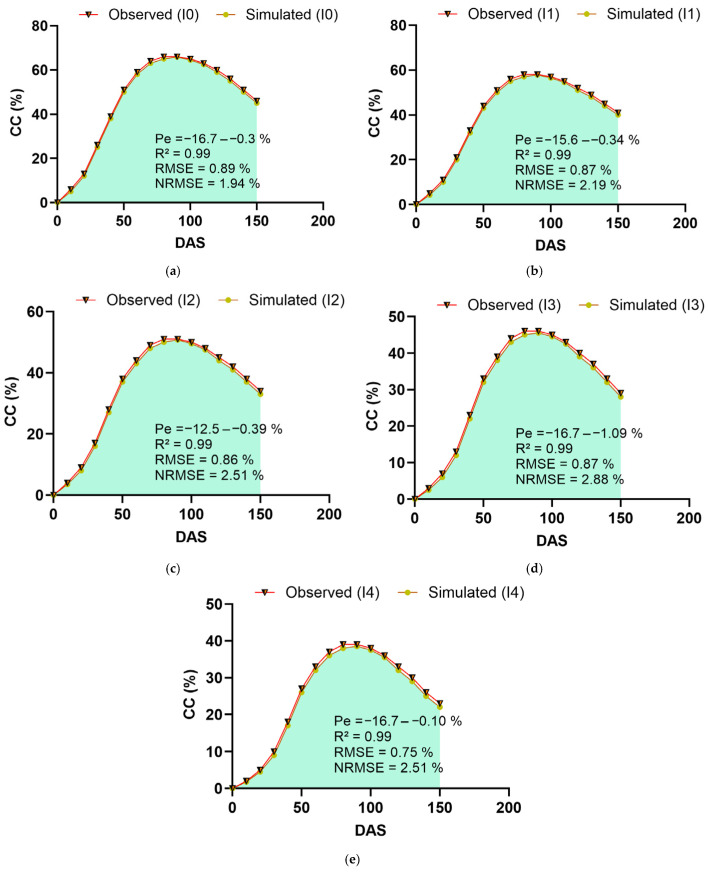
Calibration of canopy cover (CC) for winter wheat during the 2023 growing season under different salinity treatments (I0–I4). Panels (**a**–**e**) correspond to I0, I1, I2, I3, and I4, respectively. Observed and simulated CC are plotted against days after sowing (DAS). The shaded area represents the deviation between simulated and observed values.

The calibration of canopy cover (CC) was used to assess the performance of the AquaCrop model under saline irrigation conditions (I0–I4). The model showed close agreement between simulated and observed values across all salinity treatments. It reproduced CC dynamics throughout the growing cycle, from emergence to maximum cover and subsequent decline. Model performance was high, with coefficients of determination (R^2^ = 0.99) and low error metrics (RMSE = 0.75–0.89%; NRMSE = 1.94–2.88%). Prediction error (Pe) ranged from –16.70 to –0.10%, indicating a slight underestimation, more pronounced under higher salinity levels (I3 and I4). Overall, the model adequately captured canopy cover dynamics under varying salinity conditions (Figure 5).

#### 3.1.2. Simulation of Soil Water Content

**Figure 6:**
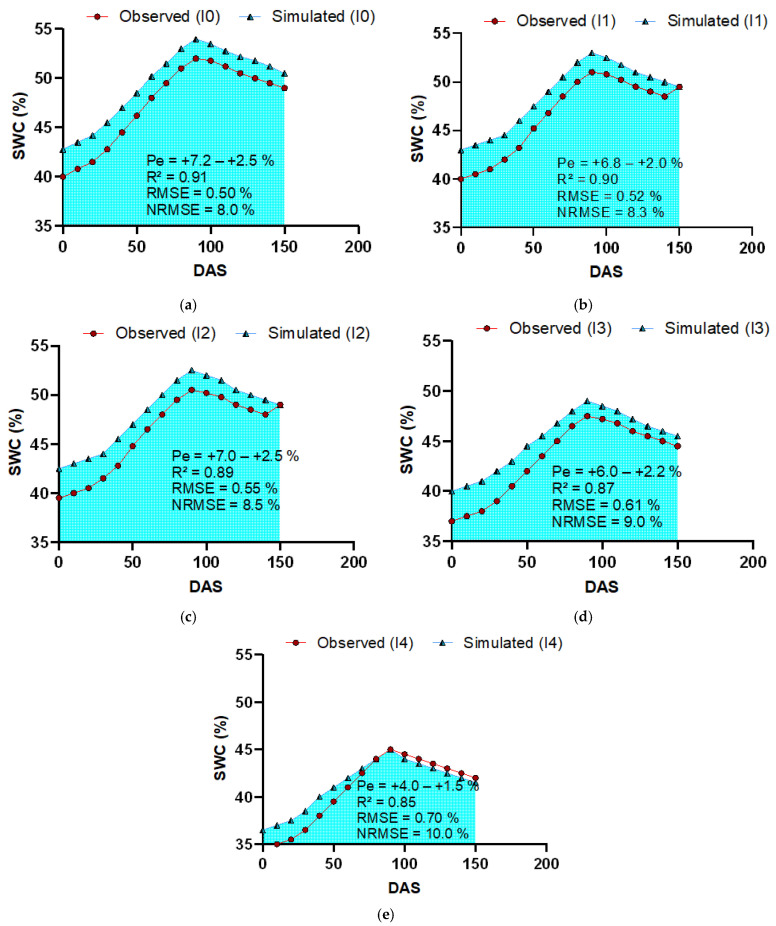
Calibration of soil water content (SWC) for winter wheat during the 2023 growing season under salinity treatments (I0–I4). Panels (**a**–**e**) correspond to I0, I1, I2, I3, and I4, respectively. Observed and simulated SWC are plotted against days after sowing (DAS). The shaded area represents the deviation between simulated and observed values.

The simulated and observed soil water content (SWC) under different salinity treatments (I0–I4) is presented in Figure 6. SWC dynamics followed similar patterns between simulated and observed values across all treatments. SWC increased during the growing period, reached a maximum, and then declined toward the end of the season. The AquaCrop model reproduced SWC dynamics with acceptable agreement, with R^2^ values ranging from 0.85 to 0.91. RMSE values varied between 0.50 and 0.70%, while NRMSE values ranged from 8.0 to 10.0%. Prediction error (Pe) ranged from +1.5% to +7.2%, indicating a slight overestimation, particularly under higher salinity levels. Model performance slightly decreased with increasing salinity, as reflected by lower R^2^ and higher error values.

#### 3.1.3. Simulation of Grain Yield and Biomass

Table 6 presents the simulated and observed grain yield (GY) and above-ground biomass (B) under different salinity treatments (I0–I4), further evaluating model performance under saline conditions. Both GY and B decreased with increasing irrigation-water salinity. Grain yield declined from 4.31 t ha^−1^ (I0) to 3.80 t ha^−1^ (I3), and further to 2.60 t ha^−1^ under I4. Similarly, biomass decreased from 14.53 t ha^−1^ (I0) to 12.90 t ha^−1^ (I3), followed by a marked reduction to 8.80 t ha^−1^ under I4. The AquaCrop model simulated GY and biomass with good accuracy. R^2^ values reached 0.98 for GY and 0.85 for biomass, with RMSE values of 0.10 and 0.25 t ha^−1^, respectively. NRMSE values remained below 3% for both variables. Overall, the model adequately captured yield and biomass responses across the salinity gradient.

#### 3.1.4. Simulation of Evapotranspiration

**Figure 7:**
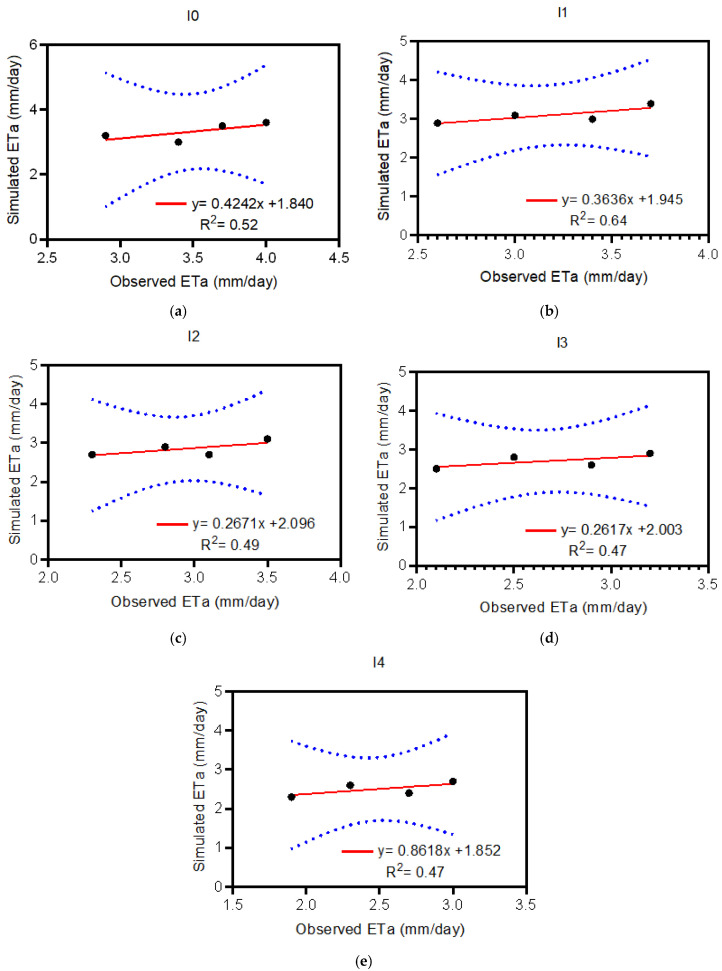
Simulation of actual evapotranspiration (ETa) for winter wheat during the 2023 growing season under irrigation treatments (I0–I4). Panels (**a**–**e**) correspond to I0, I1, I2, I3, and I4. Observed ETa is plotted on the *x*-axis and simulated ETa on the *y*-axis. The solid line represents the linear regression between observed and simulated values, with the corresponding equation and R^2^ indicated in each panel. The dotted curves represent the dispersion around the regression line.

Actual evapotranspiration (ETa) decreased consistently with increasing irrigation-water salinity, from 3.50 mm day^−1^ under I0 to 2.48 mm day^−1^ under I4, corresponding to an overall reduction of 29.30%. Intermediate treatments followed a gradual decline, confirming a consistent response of ETa to the salinity gradient. The AquaCrop model reproduced this pattern with moderate performance, as indicated by coefficients of determination (R^2^) ranging from 0.47 to 0.65. The highest agreement was observed under I1 (R^2^ = 0.65), whereas lower values under higher salinity levels (I3–I4; R^2^ ≈ 0.47–0.50) reflect increased variability between simulated and observed ETa under stress conditions. Under I0, the model showed moderate agreement (R^2^ = 0.52) (Figure 7).

#### 3.1.5. Validation of AquaCrop Model Results 

##### Canopy Cover Validation Results

**Figure 8:**
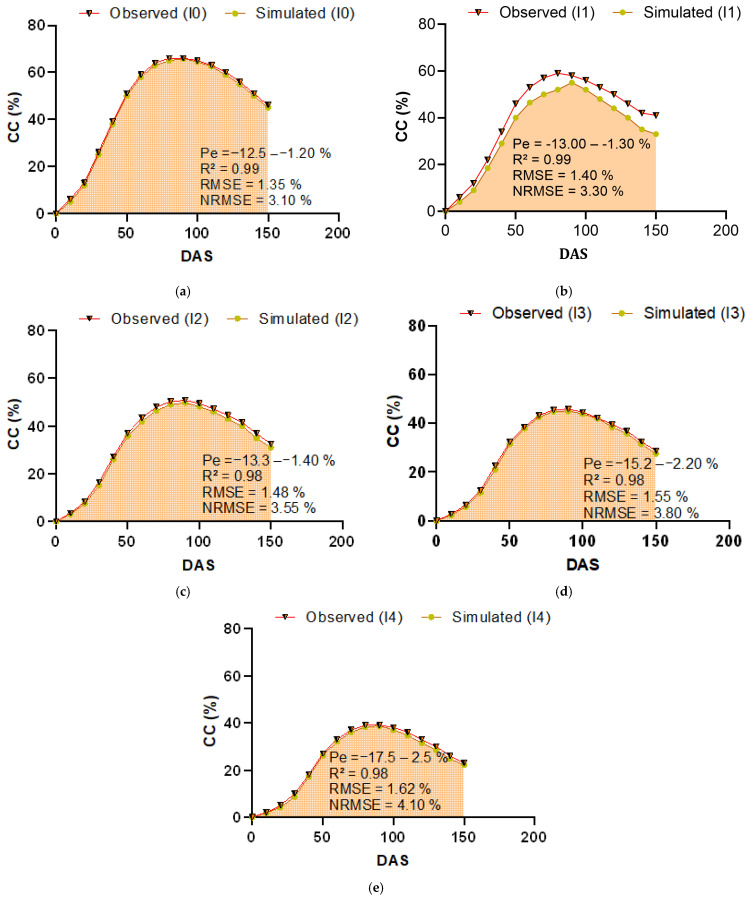
Validation of canopy cover (CC) for winter wheat during the 2022 growing season under salinity treatments (I0–I4). Panels (**a**–**e**) correspond to I0, I1, I2, I3, and I4, respectively. Observed and simulated CC are plotted against days after sowing (DAS). The shaded area represents the deviation between simulated and observed values.

The canopy cover (CC) validation results are presented in Figure 8. Simulated and observed values showed close agreement across all salinity treatments. Coefficients of determination remained high (R^2^ = 0.96–0.98), indicating a strong linear relationship. The index of agreement (d = 0.97–0.99) confirmed the consistency of model performance during the validation period. Error metrics were slightly higher than during calibration, with RMSE values ranging from 1.35 to 1.62% and NRMSE values between 3.10% and 4.10%. Prediction error (Pe) ranged from –17.5% to –12.5%, indicating a slight underestimation, more pronounced under higher salinity levels.

##### Soil Water Content Validation Results

**Figure 9:**
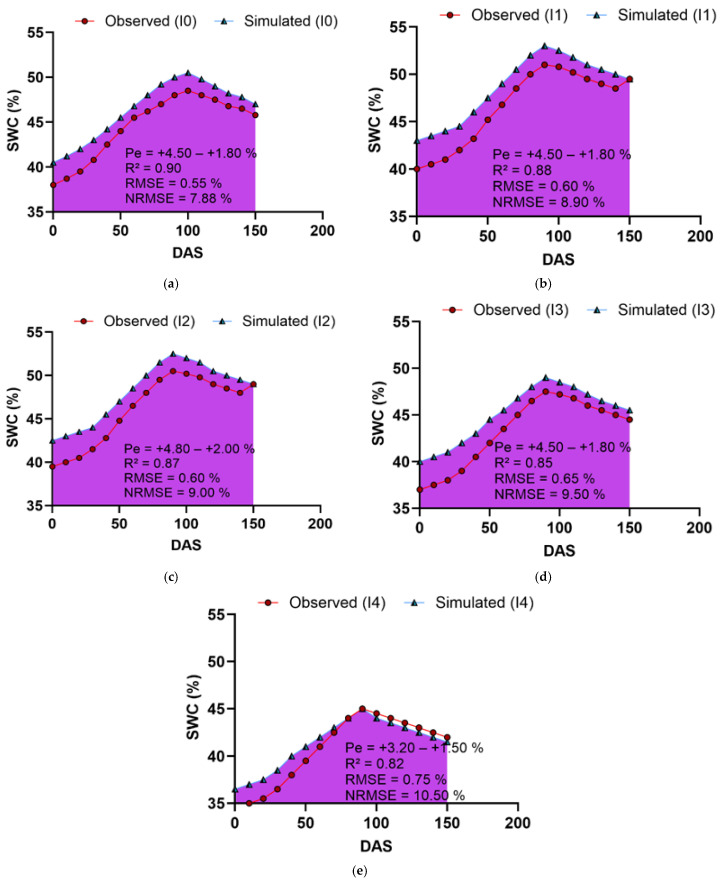
Validation of soil water content (SWC) for winter wheat during the 2022 growing season under salinity treatments (I0–I4). Panels (**a**–**e**) correspond to I0, I1, I2, I3, and I4, respectively. Observed and simulated SWC are plotted against days after sowing (DAS). The shaded area represents the deviation between simulated and observed values.

The validation results for soil water content (SWC) are presented in Figure 9. The AquaCrop model reproduced SWC dynamics across all salinity treatments (I0–I4), with similar temporal patterns between simulated and observed values. Coefficients of determination ranged from 0.82 to 0.90, indicating a satisfactory correspondence. Error metrics remained moderate, with RMSE values between 0.55 and 0.75% and NRMSE values ranging from 7.88 to 10.50%. Prediction error (Pe) ranged from +3.20% to +4.80%, indicating a slight overestimation of SWC across treatments, more noticeable under higher salinity levels.

##### Validation of Grain Yield and Biomass

The validation results for grain yield (GY) and above-ground biomass (B) are presented in Table 7. Both observed and simulated values showed a decreasing trend with increasing irrigation-water salinity (I0–I4). Grain yield declined from 4.36 t ha^−1^ (I0) to 3.90 t ha^−1^ (I3), and further to 3.10 t ha^−1^ under I4, corresponding to a reduction of approximately 29% relative to the control. A similar pattern was observed for biomass, which decreased from 14.80 t ha^−1^ (I0) to 13.30 t ha^−1^ (I3), followed by a marked decline to 10.40 t ha^−1^ under I4. Model performance remained satisfactory, with NRMSE values below 4% and R^2^ values above 0.90 for GY, and NRMSE below 3% and R^2^ above 0.80 for biomass. Overall, the model adequately captured yield and biomass responses under validation conditions.

##### Evapotranspiration Validation Results

**Figure 10:**
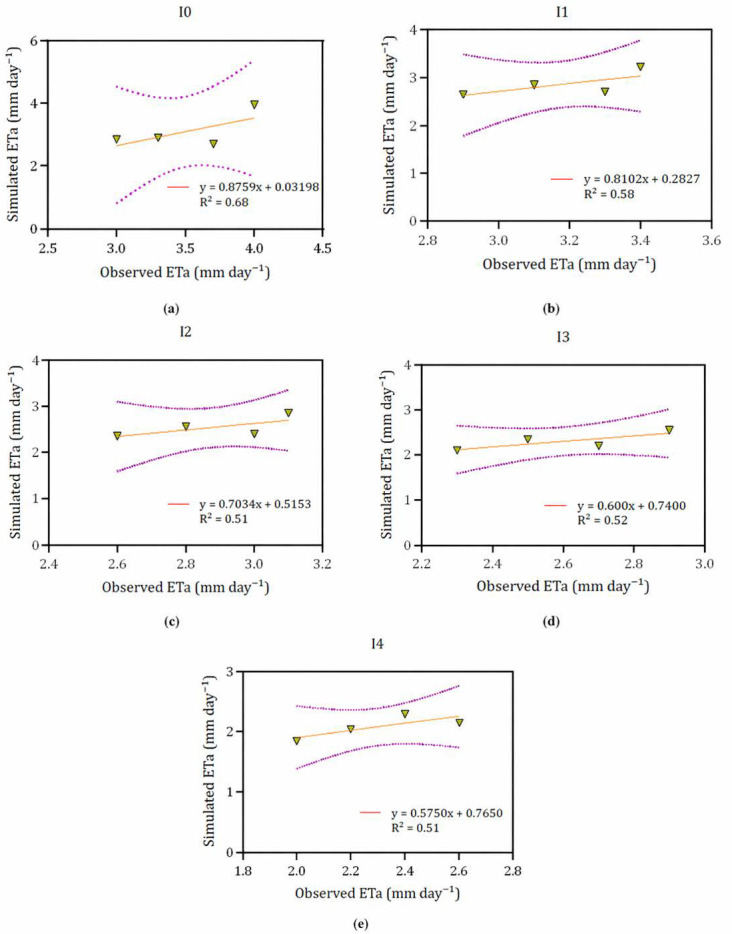
Validation of actual evapotranspiration (ETa) for winter wheat during the 2022 growing season under irrigation treatments (I0–I4). Panels (**a**–**e**) correspond to I0, I1, I2, I3, and I4, respectively. Observed ETa is plotted on the x-axis and simulated ETa on the y-axis. The solid line represents the fitted linear regression, while the dotted curves indicate the dispersion around the regression line.

The actual evapotranspiration (ETa) showed a consistent decline with increasing irrigation-water salinity, as shown in Figure 10. Observed values decreased from 3.50 mm day^−1^ (I0) to 2.55 mm day^−1^ (I4), a trend that was closely reproduced by the model. Simulated ETa followed the same pattern, although with a systematic underestimation across treatments, particularly under higher salinity levels. The AquaCrop model demonstrated moderate performance in simulating ETa during the validation phase, with R^2^ values ranging from 0.51 to 0.68. Higher agreement under non-saline conditions (I0; R^2^ = 0.68) and lower values under saline treatments (I1–I4; R^2^ = 0.51–0.58) indicate increased variability in ETa responses under stress conditions. The consistent underestimation, supported by regression slopes below unity (0.58–0.88), highlights a systematic deviation between simulated and observed values.

### 3.2. Irrigation Salinity Management Scenarios

**Figure 11:**
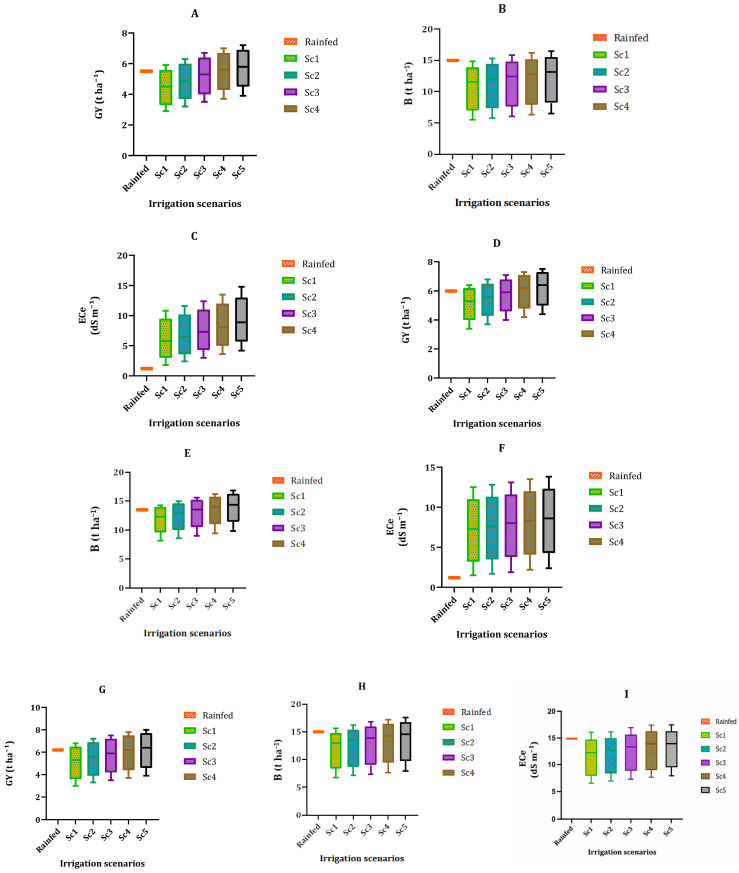
Estimated grain yield (GY), biomass (B), and soil electrical conductivity (ECe) under different saline irrigation scenarios (1996–2007 and 2018–2023). Panels (**A**–**C**), (**D**–**F**), and (**G**–**I**) represent dry, normal, and wet years, respectively.

The irrigation scenarios (Sc1–Sc5) and irrigation-water salinity levels (I0–I6), as defined in Section 2.2, represent increasing irrigation intensity and salinity levels, respectively. Simulation results under these conditions, combined with contrasting climatic periods (1996–2007 and 2018–2023), are presented in Figure 11. Under dry conditions (Figure 11A–C), grain yield (GY) decreased markedly with increasing irrigation-water salinity, declining from about 6.2 t ha^−1^ under low salinity to around 4.1 t ha^−1^ under high salinity. Biomass (B) showed a similar decline, while soil electrical conductivity (ECe) increased to approximately 10.3 dS m^−1^. The reduction in GY and B became more pronounced beyond the threshold of approximately 7.0 dS m^−1^. Under normal climatic conditions (Figure 11D–F), GY remained higher, ranging from approximately 6.6 t ha^−1^ under low salinity to about 4.5 t ha^−1^ under high salinity. Biomass followed the same trend, while ECe increased with salinity and irrigation intensity, reaching values in the range of 9.8–11.2 dS m^−1^. Under wet climatic conditions (Figure 11G,H), GY reached its highest values, around 7.6 t ha^−1^ under low salinity, and decreased to approximately 4.6 t ha^−1^ under higher salinity. Biomass showed a similar response, whereas ECe increased further, reaching approximately 11.5–13.2 dS m^−1^ under high salinity and irrigation intensity.

### 3.3. Correlation of Grain Yield, Total Biomass, and Soil Salinity with Saline Irrigation Treatments Based on Irrigation Scenarios

**Figure 12:**
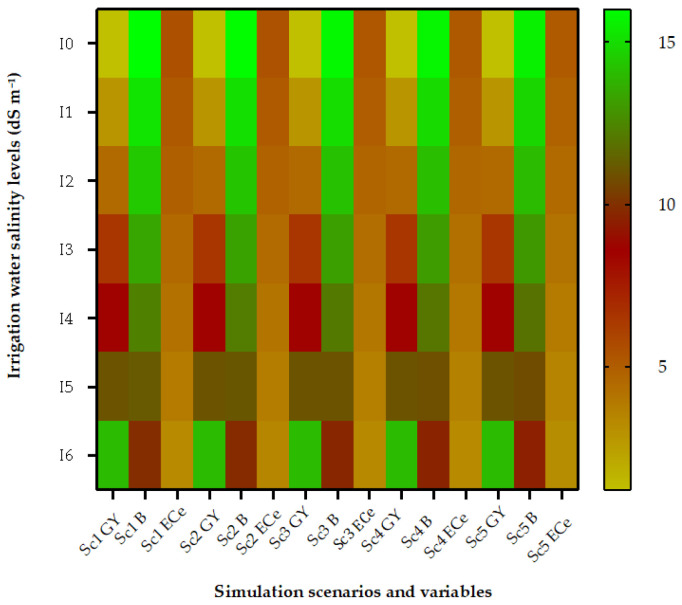
Heatmap illustrating relationships between grain yield (GY), above-ground biomass (B), and soil electrical conductivity (ECe) across irrigation-water salinity levels (I0–I6) and simulation scenarios (Sc1–Sc5).

The combined effects of irrigation-water salinity and irrigation intensity (Sc1–Sc5) on grain yield (GY), biomass (B), and soil electrical conductivity (ECe) are shown in Figure 12. The salinity range includes both the experimental treatments (1.5–9 dS m^−1^; I0–I4) and additional simulated levels (10–12 dS m^−1^; I5–I6) introduced to extend the analysis beyond experimental conditions. GY and B decreased consistently with increasing salinity, whereas ECe increased across all irrigation scenarios. Reductions in yield and biomass remained limited up to approximately 7 dS m^−1^, particularly under low to moderate irrigation intensities (Sc1–Sc3). Beyond this level, a marked decline in GY and B was observed, especially under higher irrigation intensities (Sc4–Sc5). ECe increased with both salinity and irrigation intensity, with the highest values observed under Sc4 and Sc5, while lower irrigation levels (Sc1–Sc2) maintained comparatively lower soil salinity.

### 3.4. Impact of Saline Irrigation on Soil Profile Moisture and Salinity

**Figure 13:**
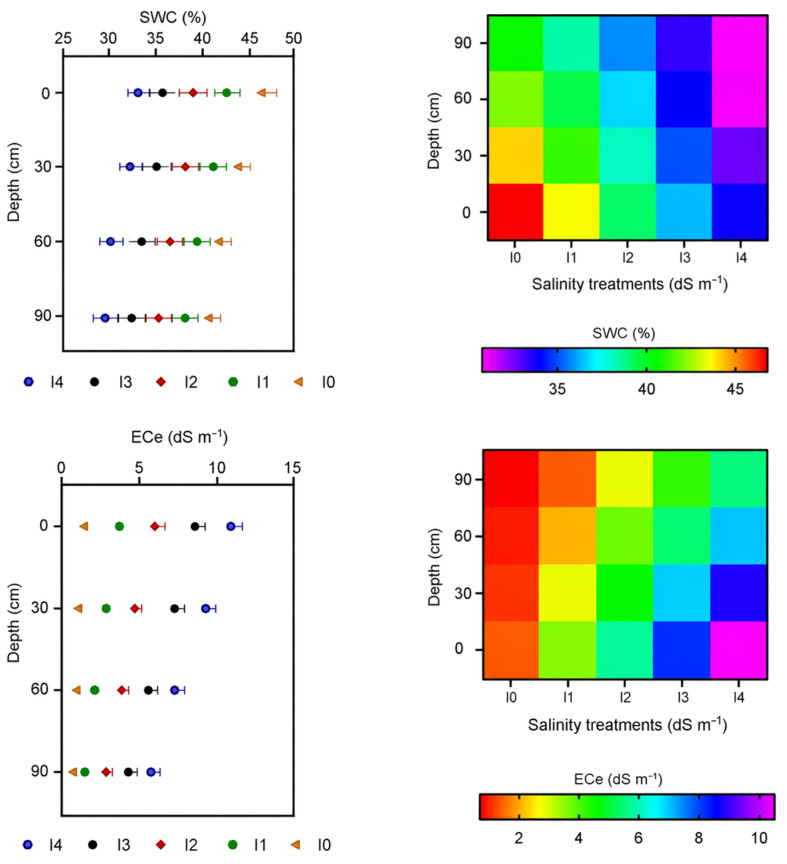
Vertical distribution of soil water content (SWC) and soil electrical conductivity (ECe) under saline irrigation treatments (I0–I4).

The vertical distribution of soil water content (SWC) and soil electrical conductivity (ECe) under salinity treatments (I0–I4) is shown in Figure 13. SWC decreased consistently with increasing irrigation-water salinity across all soil depths, with more pronounced reductions in the upper layer (0–30 cm), where values ranged from 46–48% (I0) to 33–35% (I4). At greater depths (60–90 cm), SWC ranged from 30 to 42%, with less variation across treatments. In contrast, ECe increased markedly with salinity and decreased with depth. Surface ECe values ranged from 1–2 dS m^−1^ (I0) to 9–10 dS m^−1^ (I4), while values at 90 cm ranged from 2 to 6 dS m^−1^.

## 4. Discussion

The AquaCrop model demonstrated a strong ability to reproduce canopy development under saline irrigation conditions in the Tadla Plain. The close agreement between simulated and observed canopy cover (CC), reflected by high coefficients of determination and low error metrics, indicates that the model accurately captured both the magnitude and temporal dynamics of canopy expansion and senescence across salinity treatments. This performance highlights the robustness of the calibrated canopy growth (CGC) and canopy decline (CDC) parameters in representing wheat responses to salinity stress. Under semi-arid conditions, salinity reduces canopy development through osmotic effects that limit leaf expansion and ionic toxicity that accelerates senescence, ultimately constraining photosynthetic activity and growth [66,67,68]. The model’s ability to reproduce the observed decline in CC with increasing salinity confirms that these physiological responses are adequately represented within its stress-response framework. Given the central role of canopy cover in controlling transpiration, radiation interception, and biomass accumulation, its accurate simulation is essential for linking crop growth to water use. These results are consistent with previous studies demonstrating the reliability of AquaCrop in simulating canopy development under water-limited and saline conditions [38,69]. Despite this overall strong performance, a slight underestimation of CC was observed under higher salinity levels. This deviation likely reflects the sensitivity of canopy development to short-term environmental variability under combined salinity and high evaporative demand. Rapid physiological changes during senescence may not be fully captured by the model’s simplified stress functions. Similar discrepancies have been reported under severe stress conditions, where model performance tends to decrease due to increased environmental variability [70,71].

Simulated grain yield (GY) and above-ground biomass (B) declined consistently with increasing irrigation-water salinity, reflecting the combined effects of osmotic stress, ion toxicity, and reduced photosynthetic activity, as widely reported in the literature [66,72]. This response followed a non-linear threshold pattern consistent with classical salt–yield relationships [73], with relatively stable crop performance under moderate salinity and a sharp decline beyond a critical threshold. The identification of a tolerance threshold around 7 dS m^−1^ suggests that the studied cultivar can maintain growth under moderate stress through physiological regulation mechanisms such as osmotic adjustment and partial maintenance of photosynthetic activity. Beyond this threshold, the marked decline in GY and B indicates severe disruption of carbon assimilation, reduced stomatal conductance, and impaired biomass allocation, associated with salinity-induced damage to chloroplast structure and function [68]. The ability of AquaCrop to reproduce this threshold-type response demonstrates that the model effectively integrates the combined effects of salinity on canopy development, transpiration, and biomass accumulation. Similar results have been reported by Zhai et al. (2022) [25], confirming the capacity of AquaCrop to simulate wheat productivity under saline irrigation, although some discrepancies may occur under high salinity. From an agronomic perspective, these findings highlight the importance of maintaining salinity levels below the identified threshold to avoid disproportionate yield losses, in agreement with field-based studies emphasizing the role of physiological adaptation and stress regulation under saline conditions [74].

The AquaCrop model showed good performance in simulating soil water content (SWC), indicating that the main soil–plant–atmosphere processes governing soil moisture dynamics were adequately represented under saline conditions. However, a slight overestimation of SWC was observed under higher salinity levels, reflecting the interaction between salinity stress and plant water uptake. Under saline conditions, osmotic stress reduces root water absorption, leading to increased residual soil moisture, as widely reported in wheat under salt stress [75,76,77]. From a modeling perspective, this overestimation highlights limitations in representing coupled water–solute dynamics. Although AquaCrop accounts for salinity effects on crop transpiration, it simplifies soil heterogeneity and solute transport processes, which are critical for water retention and redistribution. Similar findings were reported by Zhai et al. (2022) [25], who observed an overestimation of soil moisture under saline conditions. In addition, salinity-induced physiological responses, including reduced transpiration and altered plant water use, further influence soil moisture dynamics by limiting water extraction from the soil profile [78]. These complex interactions, combined with environmental variability, explain the slight decline in model performance under higher salinity levels. Comparable trends have been reported in other AquaCrop applications, where model accuracy decreases under severe stress due to simplified representations of stress interactions [79,80].

In contrast to other simulated variables, the performance of the AquaCrop model in reproducing actual evapotranspiration (ETa) remained moderate, as reflected by lower R^2^ values (0.47–0.65) compared to canopy cover (CC), soil water content (SWC), and grain yield (GY). This limitation is primarily related to the inherent complexity of evapotranspiration processes governed by tightly coupled soil–plant–atmosphere interactions. Under the semi-arid conditions of the Tadla Plain, characterized by high evaporative demand and strong thermal variability, ETa responds rapidly to short-term changes in soil moisture and salinity stress. Stomatal conductance is particularly sensitive to combined water and osmotic stress, leading to rapid fluctuations in transpiration rates. However, AquaCrop represents these responses using empirical stress coefficients that smooth temporal variability. As a result, the model captures general seasonal trends but may fail to accurately reproduce ETa dynamics under saline conditions. Soil-related factors further contribute to these discrepancies. Salinity reduces soil water potential and alters hydraulic properties, limiting root water uptake and modifying the partitioning between soil evaporation and plant transpiration. These coupled water–salt interactions are only partially represented in AquaCrop, which simplifies soil processes to maintain model robustness. Previous studies have similarly reported reduced model performance in simulating ETa under stress conditions due to simplified representations of soil water–salt dynamics [81,82,83,84].

Moreover, irrigation practices under saline conditions introduce additional feedback mechanisms. While irrigation may temporarily sustain evapotranspiration, it can also promote salt accumulation in the root zone, which restricts plant water uptake and alters ETa dynamics over time. These interactions between irrigation, salinity, and crop water use are difficult to fully capture using simplified modeling approaches and have been identified as key challenges in saline environments [85,86,87]. This limitation is consistent with the conceptual design of AquaCrop, which prioritizes robustness and simplicity over detailed representation of complex physiological and soil processes. Despite these constraints, the moderate performance of ETa simulations did not significantly affect the accuracy of biomass (B) and grain yield (GY) predictions. This is because AquaCrop links biomass production primarily to cumulative transpiration rather than daily ETa dynamics. Consequently, even if short-term fluctuations are not fully captured, the model remains capable of providing reliable estimates of seasonal crop productivity under saline conditions.

The relationship between evapotranspiration (ETa), biomass production, and grain yield is a fundamental component of the AquaCrop modeling framework [37,38]. In this model, biomass is driven by crop transpiration through normalized water productivity (WP), while grain yield is subsequently determined by the harvest index (HI). In this context, uncertainties in ETa may propagate to biomass and ultimately to grain yield. However, in the present study, ETa showed moderate agreement with observed values (R^2^ ranging from 0.47 to 0.65 during calibration and from 0.51 to 0.68 during validation), whereas biomass and grain yield were simulated with high accuracy (R^2^ up to 0.85 and 0.98 during calibration and remaining above 0.80 and 0.92 during validation, respectively, with NRMSE below 4%). Despite an approximate 29% reduction in ETa under high salinity conditions, the model accurately reproduced the corresponding decrease in biomass and grain yield across treatments. This comparison indicates that ETa uncertainty, although relatively high, results in only minor errors in grain yield prediction (NRMSE < 4%), demonstrating a limited propagation of ETa uncertainty to seasonal yield. This reflects an attenuation of uncertainty from ETa to yield at the seasonal scale. This behavior can be explained by the model structure, where biomass depends on cumulative transpiration rather than instantaneous ETa values, and the harvest index (HI) acts as a regulating factor that buffers the effect of ETa variability on final yield estimation [88].

The multi-scenario analysis indicates that crop response to salinity is governed by the combined effects of irrigation intensity, soil salinity dynamics, and climatic conditions rather than salinity alone. Increasing irrigation-water salinity consistently reduced GY and B; however, the magnitude of this reduction varied across climatic conditions. A quantitative comparison further highlights this interaction. Under low salinity, GY under wet conditions was approximately 13.15% and 18.42% higher than under normal and dry conditions, respectively, confirming the strong role of water availability in alleviating osmotic stress. Under high salinity, this difference decreased to about 10.86%, indicating that the mitigating effect of favorable climatic conditions becomes less pronounced as salinity intensifies. A similar but less pronounced pattern was observed for B, with increases of approximately 6.74% and 17.20% under wet conditions compared with normal and dry conditions, respectively, while differences became negligible under high salinity conditions. Climatic variability therefore modulates crop response primarily under low to moderate salinity, where adequate soil moisture sustains plant water uptake and partially offsets osmotic stress, as also reported in previous studies [89,90]. In contrast, under high salinity, crop performance becomes increasingly controlled by salinity-induced limitations, reducing the relative influence of climatic conditions. Despite improved crop performance under wet conditions, ECe increased by approximately 19.4% compared with dry conditions under high salinity. This indicates that enhanced yield and biomass were not associated with reduced soil salinity, but rather with improved water availability. These findings emphasize a key trade-off: increasing irrigation can sustain crop productivity in the short term, but may accelerate salt accumulation in the soil profile, particularly under semi-arid conditions [88,91].

High irrigation intensity accelerated salt accumulation in the root zone, indicating that excessive irrigation under saline conditions enhances evapoconcentration and reduces long-term productivity [92]. The identification of a threshold around 7 dS m^−1^ highlights a critical transition in crop response: below this level, plants maintain growth through osmotic adjustment, whereas above it, reduced soil water potential and ionic stress sharply limit biomass production. These results confirm that irrigation acts as both a buffering and aggravating factor depending on its management. Soil salinity dynamics further explain these responses, as the vertical distribution of ECe reflects the balance between upward evaporative fluxes and limited leaching, leading to salt accumulation in surface layers under semi-arid conditions [85]. At the same time, reduced soil water content (SWC) under salinity indicates decreased root water uptake and enhanced soil evaporation, illustrating strong soil–plant interactions under combined salinity and water stress [93]. From a modeling perspective, the ability of AquaCrop to reproduce these coupled soil–plant–water interactions across irrigation scenarios and contrasting climatic periods demonstrates its robustness for multi-year analysis. These findings have direct implications for irrigation management, emphasizing that maintaining salinity below the identified threshold and avoiding excessive irrigation are essential to improve water-use efficiency while limiting long-term soil salinity risks.

## 5. Conclusions

This study demonstrates the value of the AquaCrop model as a reliable decision-support tool for managing saline irrigation of winter wheat under semi-arid Moroccan conditions. The model was successfully calibrated (2023) and independently validated (2022), accurately reproducing key crop and soil processes, including canopy development, soil water content, evapotranspiration, biomass, yield, and soil salinity dynamics, confirming the robustness of the calibrated parameters for the Achtar variety. The results reveal a clear threshold response to salinity. Crop productivity remained stable under low to moderate salinity, with a critical threshold identified around 7 dS m^−1^, beyond which yield declined sharply. Scenario analysis further demonstrated that irrigation management strongly controls salinity effects: moderate irrigation maintained yield while limiting salt accumulation, whereas higher irrigation inputs accelerated soil salinization without improving productivity. These findings provide quantitative thresholds and practical irrigation management guidelines for Moroccan conditions, where such references remain limited. The ability of AquaCrop to reproduce crop–soil–water interactions under contrasting climatic conditions supports its use for multi-year simulations and irrigation planning. Overall, sustainable management under saline irrigation requires an integrated approach that considers crop response, soil processes, and climate variability. Future research should focus on long-term field validation and the integration of economic and environmental indicators to support informed decision-making.

## Figures and Tables

**Table 1:** Soil textural, chemical, and hydraulic properties of the experimental field used for model calibration and validation. FC: Field capacity; PWP: Permanent wilting point; Ksat: Saturated hydraulic conductivity.

Soil Layer Depth (cm)	Particle Size Distribution (%)						Soil Texture	
	Sand (%)		Silt (%)		Clay (%)			
0–30	37.75		33.18		28.9		Clay Loam	
30–60	37.9		33.82		28.1		Clay Loam	
60–90	36.3		31.41		29.4		Clay Loam	
**Chemical parameters**								
	**pH**	**ECe** **(dS m^−1^)**	**OM** **(%)**	**Total CaCO_3_** **(%)**	**NH_4_^+^** **(mg kg^−1^)**	**NO_3_^−^** **(mg kg^−1^)**	**Total mineral N**	**CEC** **(cmolc kg^−1^)**
0–30	8.36	0.21	1.62	15.85	15.37	34.65	50.02	45.3
30–60	8.45	0.22	1.33	9.85	13.65	28.00	41.65	42.2
60–90	8.50	0.18	0.86	13.8	10.10	18.90	29.00	41.4
**Physical and hydraulic parameters**								
	**Bulk Density** **(g cm^−3^)**		**Ksat** **(mm day^−1^)**	**Saturation** **(cm^3^ cm^−3^)**	**FC** **(cm^3^ cm^−3^)**		**PWP** **(cm^3^ cm^−3^)**	
0–30	1.40		125	0.50	0.39		0.23	
30–60	1.46		500	0.50	0.30		0.20	
60–90	1.52		500	0.50	0.30		0.21	

Soil texture classification follows the United States Department of Agriculture (USDA) system [57].

**Table 2:** Agronomic and technological characteristics of the wheat variety Achtar. GW: Grain Weight; TW: Test Weight; PC: Protein Content; ZI: Zeleny Index; MQ: Milling Quality.

Wheat Variety	Adaptation Zone	Average Yield (t ha^−1^)	Potential Yield (t ha^−1^)	Plant Height (cm)	Tillering Capacity	Maturity Cycle	Lodging Resistance	Technological Traits
Achtar	Recommended for fertile soils and irrigated conditions	4.5	11.8 (irrigated); 11.2 (rainfed)	75–115	Medium	Semi-early to semi-late	Good	GW: 39.18 mg TW: 81 Kg hl^−1^ PC: 13.4% ZI: 33 mL MQ: Good

**Table 3:** Summary of crop management practices, fertilization schedule, and phytosanitary treatments for winter wheat during the validation (2022) and calibration (2023) growing seasons.

Parameter	Validation	Calibration	Notes
Cultivar	Achtar	Achtar	Same cultivar both years
Growing season	Winter	Winter	—
Sowing date	24 November 2021	24 November 2022	—
Seeding rate	300 seeds m^−2^	300 seeds m^−2^	Uniform across plots
Basal P fertilization	200 kg ha^−1^ TSP	Same	At sowing
Basal K fertilization	100 kg ha^−1^ K_2_SO_4_ (45% K_2_O)	Same	At sowing
Total N fertilization	150 kg ha^−1^	150 kg ha^−1^	Split into 3 applications
-N at sowing	40% as ammonium sulfate	Same as validation	60 kg N ha^−1^
-N at tillering	20% as ammonium nitrate	Same as validation	30 kg N ha^−1^
-N at stem elongation	40% as ammonium nitrate	Same as validation	60 kg N ha^−1^
Herbicide	Lintur (1 pack ha^−1^)	Same as validation	Early vegetative stage (GS 14–15)
Nematicide	Furadan (10 kg ha^−1^)	Same as validation	At sowing
Manual weeding	—	—	Stem elongation, GS 30–32)
Fungicide	Fungicide (Planète)	Same as validation	Flag leaf stage (GS 39–49)
Harvest date	Mid–late June 2022	Mid–late June 2023	Physiological maturity

**Table 4:** Chemical characteristics of irrigation water used in the study and comparison with WHO (2017) standards [58].

Parameters	Value	WHO (2017) Standards
pH	7.65	6.5–8.5
EC	1.20	1 dS m^−1^
Ca^2+^	3.03	75 mg L^−1^
Mg^2+^	2.34	50 mg L^−1^
Na^+^	16.97	200 mg L^−1^
K^+^	0.12	10 mg L^−1^
Cl^−^	16.15	250 mg L^−1^
SO_4_^2−^	1.89	250 mg L^−1^
HCO_3_^−^	3.81	120 mg L^−1^
NO_3_^−^	5.00	50 mg L^−1^

**Table 5:** AquaCrop model parameters used for the calibration and validation of winter wheat (cv. Achtar).

Parameters	Values	Units	Determination Way
**Canopy Cover Parameters**			
Initial canopy cover (CC_0_)	5.25	%	Estimated
Maximum canopy cover (CCx)	96	%	Calibrated
Canopy growth coefficient (CGC)	0.52	% day^−1^	Calibrated
Canopy decline coefficient (CDC)	0.39	% °C day^−1^	Calibrated
**Crop parameters**			
Maximum coefficient for transpiration at CCx (Kc_Tr,x_)	1.08	–	Calibrated
Normalized crop water productivity (WP*)	16.8	g m^−2^	Calibrated
Reference harvest index (HI_0_)	44	%	Calibrated
Maximum effective rooting depth (Zr)	1.10	m	Calibrated
Minimum effective rooting depth (Zmin)	0.30	m	Model default
**Depletion of soil water thresholds**			
Leaf expansion, upper threshold (P_exp,upper_)	0.20	–	Model default
Leaf expansion, lower threshold (P_exp,lower_)	0.65	–	Model default
Stomatal closure, upper threshold (P_sto,upper_)	0.65	–	Model default
Upper threshold for canopy senescence (P_sen,upper_)	0.70	–	Model default
**Soil water balance parameters**			
Curve number (CN)	72	–	Model default
Readily evaporable water (REW)	11	mm	Model default
**Phenological parameters**			
Time to emergence	150	°C d	Observed
Time to maximum canopy	1197	°C d	Observed
Time to maximum rooting depth	864	°C d	Calibrated
Time to start of senescence	1700	°C d	Observed
Time to maturity	2800	°C d	Derived from field observations
Time to flowering	1250	°C d	Observed
Duration of flowering	200	°C d	Observed
Length of HI build-up	1100	°C d	Calibrated
**Salinity stress parameters**			
Lower threshold for salinity stress	5	dS m^−1^	Calibrated
Upper threshold for salinity stress	18	dS m^−1^	Calibrated
Canopy response to salinity	25	–	Model default
Stomatal closure response to salinity	115	–	Model default

**Table 6:** Statistical evaluation of observed and simulated grain yield (GY) and above-ground biomass (B) of winter wheat under different saline irrigation treatments (I0–I4) during the 2023 growing season. The table presents the observed and simulated mean values, percentage difference (%), root mean square error (RMSE, t ha^−1^), normalized RMSE (NRMSE, %), and the coefficient of determination (R^2^), providing a quantitative assessment of model performance.

Treatment (dS m^−1^)		Observed	Simulated		Observed	Simulated	
		GY (t ha^−1^)			B (t ha^−1^)		
I0		4.31	4.28		14.53	14.45	
I1		4.12	4.05		13.95	13.80	
I2		3.95	3.88		13.30	13.10	
I3		3.80	3.75		12.90	12.70	
I4		2.60	2.55		8.80	8.60	
**Parameter**	**Unit**	**Observed** **mean**	**Simulated mean**	**Percentage difference** **(%)**	**RMSE** **(t ha^−1^)**	**NRMSE** **(%)**	**R^2^**
GY	t ha^−1^	3.76	3.70	1.60	0.10	2.70	0.98
B	t ha^−1^	12.30	12.13	1.40	0.25	2.00	0.85

**Table 7:** Statistical evaluation of observed versus simulated grain yield (GY) and above-ground biomass (B) of winter wheat under different saline irrigation treatments (I0–I4) during the 2022 growing season. The table presents the observed and simulated mean values, percentage difference (%), root mean square error (RMSE, t ha^−1^), normalized RMSE (NRMSE, %), and the coefficient of determination (R^2^), providing a quantitative assessment of model validation performance.

Treatment (dS m^−1^)		Observed		Simulated	Observed	Simulated	
		GY (t ha^−1^)			B (t ha^−1^)		
I0		4.36		4.30	14.80	14.55	
I1		4.22		4.15	14.30	14.00	
I2		4.05		3.98	13.80	13.55	
I3		3.90		3.82	13.30	13.05	
I4		3.10		2.95	10.40	10.10	
**Parameter**	**Unit**	**Observed mean**	**Simulated mean**	**percentage difference** **(%)**	**RMSE** **(t ha^−1^)**	**NRMSE** **(%)**	**R^2^**
GY	t ha^−1^	3.93	3.84	2.30	0.15	3.80	0.92
B	t ha^−1^	13.32	13.05	2.00	0.32	2.40	0.84

## Data Availability

The data presented in this study are available on request from the corresponding author. The data are not publicly available due to institutional restrictions.
